# LIFETIME EXPERIENCES OF GRIEF AMONG RECENTLY BEREAVED AFRICAN AMERICANS

**DOI:** 10.1093/geroni/igz038.1898

**Published:** 2019-11-08

**Authors:** Danielle L McDuffie, Rebecca S Allen, Sheila Black, Martha R Crowther, Ryan Whitlow, Laura Acker

**Affiliations:** The University of Alabama, Tuscaloosa, Alabama, United States

## Abstract

This study sought to investigate the ways recently bereaved African American middle to older aged adults conceptualized both prior and present loss. Fourteen African American men and women aged 46 years and older (M=62.6) completed one time, in-person semi-structured interviews detailing their grief experiences. Interview transcripts were then coded using a content analysis. Four themes were reported during prior loss (Continuing on with Normal Life/ Time, Faith/ Religion, Reminiscing/ Reminiscence, Social Support) along with present loss (Faith/ Religion, Keeping Busy, Reminiscence, Social Support). Men and women in the sample were found to cope in relatively consistent manners despite the timing of the loss, and in manners consistent with literature detailing African American grief outcomes. This information could help inform both bereaved African Americans and those seeking to aid African Americans during times of bereavement in proactively having knowledge of coping mechanisms that have been used historically and found to be beneficial.

